# Weighing on us all? Quantification and cultural responses to obesity in NHS Britain

**DOI:** 10.1177/0073275319842965

**Published:** 2019-04-24

**Authors:** Roberta Bivins

**Affiliations:** Centre for the History of Medicine, Department of History, University of Warwick, UK

**Keywords:** Body mass index, obesity, National Health Service, self-weighing, self-quantification, health citizenship

## Abstract

How do cultures of self-quantification intersect with the modern state, particularly in relation to medical provision and health promotion? Here I explore the ways in which British practices and representations of body weight and weight management ignored or interacted with the National Health Service between 1948 and 2004. Through the lens of overweight, I examine health citizenship in the context of universal health provision funded from general taxation, and track attitudes toward “overweight” once its health implications and medical costs affected a public service as well as individual bodies and households. Looking at professional and popular discourses of overweight and obesity, I map the persistence of a highly individual culture of dietary and weight self-management in postwar Britain, and assess the degree to which it was challenged by a new measure of “obesity” – the body mass index – and by visions of an NHS burdened and even threatened by the increasing overweight of the citizens it was created to serve.

## Introduction

In the last twenty years, scholars across the humanities and social sciences have paid increasing attention to the “quantified self” movement. In the process, they have brought the contemporary enthusiasm for self-measurement and other forms of quantitative self-observation into conversation with other forms of self-knowing, from diary-keeping to DNA self-testing, and with the deeper history of quantification in society and the human sciences.^1^ Such studies are often rooted in an American context, and position “self-tracking” as a marker and even precursor of the radical individualism of U.S. society.^2^ A substantial and growing literature has also engaged with the emergence of and adaptation to specifically the domestic use of technologies of exact measurement.^3^ But neither practices of self-quantification nor these measuring technologies moved into the home “naturally” or automatically. Rather, their gradual but comprehensive domestication resulted from a range of complex push and pull factors, social, economic, cultural, and political. Elsewhere, historians and sociologists have examined the roles of social and cultural norms, particularly around physical appearance and bodily performance.^4^ State and commercial interests and interventions too played a role.^5^ Medical professionals and other health advisors mediated and supported the creation of quantifiably “normal” and normative human bodies. In particular, many have been keen to encourage the collection at home of data useful not only for domestic health promotion and disease prevention activities, but for public health and medical research. As early as the end of the nineteenth century, those interested in population health, for example, prized data about individual adult weight as a potential state (and commercial) resource, though their enthusiasm was not universally shared by doctors diagnosing and treating individual patients.^6^

Here, I examine a different aspect of the emerging culture of quantified self-management: its interactions with the state, and in particular with the British postwar welfare state. Did the advent of the National Health Service (NHS), which opened its doors on the July 5^th^, 1948, produce any marked shift in British discourses of corpulence, body weight, and quantification? What about the many changes to which the system has been subject particularly since the 1970s, as marketization, individualized medicine, and what Martin Powell has called “neo-republican citizenship” displaced the older models of decommodification, social medicine, and social-democratic citizenship that shaped its birth?^7^ Where did (and do) self-measurement and self-regulation fit in the context of a national system delivering universal access to medical care, funded from general taxation, and almost entirely free at the point of delivery? Drawing on public and professional discourse around weight management between the 1948 inception of the NHS and the 2004 “Choosing Health: Making Healthy Choices Easier” White Paper, I will track attitudes toward “overweight” once its health implications and medical costs affected a public service as well as individual bodies and households.

## Fat and fitness: British responses to overweight before the NHS

Of course, state interest in the health of individual bodies, and the bodies of groups regarded as either particularly vulnerable or particularly essential to national status and security, emerged well before World War II. In Britain, as elsewhere, state attention to infant, child, and maternal health was familiar by the interwar years. So too were moral panics about male fitness and the risks of “racial” degeneration, prompted by military recruiters’ discovery of high levels of masculine debility during the Boer War and World War I.^8^ By the late 1930s, the government would sponsor a health and fitness campaign for the nation, one that targeted, among others, Orwell’s “little fat men” – sedentary middle-class males.^9^ As Ina Zweiniger-Bargielowska and Charlotte Macdonald have argued, “reducing culture” during this period certainly depicted self-care and self-control as public virtues and attributes of hygienic citizenship.^10^ For instance, in a deliberate echo of Admiral Horatio Nelson’s famous Battle of Trafalgar exhortation, Britain’s National Fitness Campaign (NFC, 1937–9) urged: “England expects every man and woman to be healthy and fit.”^11^

Unlike malnutrition, a key governmental concern in this period, obesity was most commonly represented as a lapse in individual rather than state responsibility. As Britain’s National Fitness Council was eager to assert, “no one can make another fit or take exercise for him,” nor could the inert and apathetic “rightly blame the borough council or anyone else” for their ill-health.^12^ Endorsing the NFC, George VI also spoke in terms of individuals’ “duty to ourselves and our generation,” and stressed the importance of individual “will” in a campaign which consistently allied – and often conflated – mental, moral, and physical fitness.^13^ In this respect, interwar cultural responses to corpulent bodies (whether defined as measurably “overweight” or simply perceived as “fat”) continued a longer tradition that framed obesity as the result of moral failings and weakness of character, facilitated by overwhelming and perhaps degenerative social change.^14^ Moreover, because obesity was still configured as a middle-class condition, its victims were commonly imagined and portrayed in popular culture as individuals to whom “compulsion” was “alien” and “uniformity” unattractive.^15^ Diet – and especially adult diet – was persistently understood as a matter of individual and household choice, operating within budgetary constraints. Dietary advice and interventions offered by agents of the state or charitable “do-gooders” received a lukewarm welcome, at best, from their intended beneficiaries.^16^

Perhaps as a consequence, before World War II, British approaches to obesity “emphasized conduct” rather than quantification.^17^ Whether under the guidance of a physician, or by following the popular advice literature, overweight adults might be encouraged to weigh themselves and to track the progress of their reducing regimes, but for the purposes of the state, these citizens were trusted with the complex task of judging their fitness by function and by form, rather than against a set of absolute numerical targets.^18^ In this, the British state also responded to wider medical ambivalence about the diagnostic value of precision anthropometry, the validity of statistical norms, and the normative height/weight tables they together enabled.^19^ Practicing clinicians faced individual patients ranging across the physiological and metabolic spectrum from, in the language of W. H. Sheldon’s then-popular theory, “ectomorphs” to “endomorphs,” and were intensely aware of their patients’ idiosyncratic habits of diet and activity. While often eager to find a simple and reliable tool for estimating obesity, they were, and would remain, skeptical about those available.^20^

Expert ambivalence about the scales notwithstanding, by the mid-twentieth century exact self-measurement was a familiar part of adult personal routine, at least in North American and Western Europe.^21^ In Britain, stepping onto the scales remained a common public activity, both for health maintenance and for entertainment, throughout the early decades of the NHS. In the late 1940s and early 1950s, following the unprecedented rigor of governmental dietary control during the war and the persistence of rationing until 1954, such public weighing apparently held little fear. As one commentator enthused in 1956: “The number of weighing machines on our piers and promenades and railway platforms, in chemists’ shops and fun-fairs and snack bars is as large as, if not larger than ever it was, and some of them seem, like telephone kiosks, to be permanently occupied.” Describing self-weighing as a custom “truly rooted in the hearts and lives of the people,” the author found the origins of its appeal and durability in the long-established place of weight as a metric of infant and child health: “The child who in the first months of life is cradled on the scale, whose every ounce is charted with loving care, is father to the man who waiting on a train on any platform anywhere cannot resist the lure of the weighing machine.”^22^ Notably, this account, and coverage like it elsewhere in the popular press, crafted self-weighing as both a tool of preventive self-care (“if it warns the corpulent merchant that he has put on another pound or two, well he is getting more for his money”) and of deeper self-knowledge. The “weighing machine,” concluded *The Times*, was infinitely fascinating precisely because it spoke to “that subject of inexhaustible interest – us.”^23^

## Encountering overweight: evaluating obesity in public health and general practice

If the British public returned willingly to the embrace of the weighing machine in the aftermath of the war, selling the scales to those formally charged with delivering postwar population health – local governments, schools, and the new NHS – was a less straightforward proposition. Despite rising British body weights and renewed public interest in “slimming,” undernutrition and nutrient deficiency malnutrition remained the principal targets of professional agitation and state-sponsored nutrition interventions and advice in the early NHS.^24^ These focused closely on infant, child, and maternal health.^25^ While the Ministry of Health, on behalf of the fledgling NHS, pleaded with the general population to practice self-care via campaigns focusing on the “Seven Rules of Health,” neither quantified health standards nor practices of self-measurement featured as aids or measures of healthy living in health education materials or exhibitions in this period.^26^ Indeed, although the Ministry of Health’s “The Health of the People” exhibition, designed and displayed by the Central Office of Information in 1948, mentioned the importance of “the hygiene of daily living,” and cited “excesses” (as well as poverty) as health threats, its advice for health promotion and self-cultivation was entirely free from quantification or self-surveillance practices.^27^ The nation accepted “the principle of collective responsibility” but explicitly for “individual health” and “personal health services.”^28^ And if health had thereby become “everybody’s business,” it still remained business to be transacted primarily by individuals in accordance with advice rather than centrally established targets.^29^

In contrast, local governments (in Britain known as “local authorities”), which retained responsibility for the bulk of environmental and preventive health services, the School Health Service, and local health education initiatives certainly used quantification to assess the health of their populations. A wide array of professionals delivered these services, but all operated under the purview of each area’s Medical Officer of Health (MOH, invariably a qualified doctor). Annual reports submitted by Medical Officers of Health (MOsH) and School Medical Officers across Greater London routinely tracked the heights and weights of target populations – infants and schoolchildren, for example. In the 1940s and early 1950s, these data served as markers for mapping population health and assessing the effects of the new universal availability of health services, as well as other health-supporting measures of the welfare state, from welfare feeding to improved housing. Local health officials celebrated the sharply increasing heights and weights of schoolchildren, while the (increasing) body weights of adult and elderly groups rarely figured.^30^

However, when rationing ended in the mid-1950s, doctors, the medical and lay press, and the Ministry of Health resumed their interrupted discussions about obesity and overnutrition. In relation to public health, these discussions initially centered around the classic “vulnerable groups” who were routinely subject to higher levels of surveillance: infants, children, and sometimes the elderly. From the mid-1950s, obesity among such populations began to attract official notice in the annual reports of Medical Officers of Health based in and around the conurbation of London.^31^

The tone of such reports changes markedly over this time, as the prevalence of quantified overweight, especially in children and adolescents, escalated. At first, few reports expressed significant concern about the expanding British body, child or adult. In 1949 and 1951, even a single case of “gross obesity” attracted attention (but not sanction) in Leyton.^32^ In Walthamstow, Dr. Elchon Hinden, Pediatrician to Whipps Cross Hospital, worried more about the effects of teasing on chubby children’s mental health than about the excess weight itself.^33^ In 1956, Croydon’s MOH, S. L. Wright, expressed frank “satisfaction” in the increasing heights and weights of children in his district. He cheerfully dismissed as “gloomy forecasts … contrary to common sense” any suggestions that such growth might be detrimental.^34^ Only in hypothetical terms would Wright concede “a developing need to watch for unnecessary obesity.”^35^ A year later, this grudging concession to affluence gained some official sanction when the UK government revitalized its expert committee on medical aspects of food policy with a remit to explore, among other things, the possible relationship between diet and heart disease in adult men.^36^

Overall, undernutrition remained for many MOsH by far the greater danger both to health and to civic society. John Maddison, MOH for Twickenham, for example, was acutely aware of the health dangers of rising obesity: “If overnutrition and obesity continues it will tend to shorten … lives.” However, for Maddison, the acknowledged physical dangers of increasing diabetes, heart disease, and accidents paled before the moral and social impact of hunger: a “contented mind needs a well-filled body. In conditions of undernutrition, people become restless and their standard of behaviour falls … I wonder if this wave of crime which we have seen this few years, especially among juveniles, is not the result of lowered moral standards from food scarcity which we went through during the war years.”^37^ “It is a sobering thought,” he added, “if a period of starvation leads to a generation of criminals.”

These worries did not deter Maddison from actively publicizing the changing face of malnutrition, especially as the state lifted its imposed dietary constraints. In the same 1952 report, he directly asked his readers: “ARE YOU FAT OR THIN?… Now that we see the end of rationing in sight, my thoughts have turned to the effects of food on the body, and to the question of how much or how little food is good for us.” As he observed, while undernutrition had always attracted the attention of medical officers, “overnutrition” too was rapidly coming under expert scrutiny. Even in his own borough, the data indicated rising mortality linked to obesity: “fat people tend to develop high blood pressure and to die earlier of heart disease and stroke… heart disease and stroke is becoming commoner as the cause of death after middle age.”^38^ Tellingly, determinations of “fatness” depended at least in part on exact measurement of weight.

Drawing on the latest nutrition research by biochemist and physiologist Robert McCance, Maddison was sympathetic to his heavier constituents, acknowledging that even very slight deviations from “energy balance” – “as little as one-thirtieth of an ounce per day” – could result in overweight.^39^ Rather than blaming them, he blamed “a civilised world with plenty to eat.”^40^ Nonetheless, for Maddison, adults’ weight was ultimately determined by personal choices: “Only one thing determines whether a person shall be fat or thin, and that is the amount of food he eats.”^41^ Thus, like the “Health of the People” exhibition of 1948, his account positioned overweight as a “subject … of outstanding interest to us as a community,” but also as one primarily for individual action: “your weight, so to speak, is in your own hands.”^42^ This was a call for active health citizenship instead of and as opposed to direct state intervention.^43^ As Charlotte Macdonald has argued in relation to the interwar fitness movement, threats to health that arose from individual choices – to be active or inert; to eat moderately or to excess – might concern the state, but the fight against them was not and could not be “in the hands” of experts, the state, or even the new NHS. This “strong line of separation drawn between governments and healthy bodies” survived the deviation of wartime nutritional interventionism. Personal volition returned to the fore in cultural models of health maintenance, and as we will see below, discourses of self-weighing in relation to adults reflected this through the persistence of its associations with choice, willpower, and the individual.^44^

By 1965, however, such laissez faire attitudes toward overweight and obesity, at least in young children and adolescents, were changing.^45^ S. L. Wright continued to ignore issues of adult weight and overweight in his annual MOH reports. However, writing in his capacity as Principal School Health Officer, Wright noted that overweight in children was “causing increasing concern.” The roots of such “concern” lay in new research that confirmed links between child and adult obesity: “overweight children become overweight adults and the risks to health which the latter experience have long been known.” Notably, these were claims and views that Wright had himself had rejected as recently as 1957, then sanguine that “the advantages of having the average child taller and heavier far exceeded the risk of some being overweight … or the theoretical dangers forecast for later life.”^46^

Working at the chalkface, Phyllis Gibbons, a Croydon School Medical Officer, knew that the focus of her efforts had to change: “[a]t the inception of the School Health Service the nutritional problems encountered by the Medical Officer were predominantly those of malnutrition.” However, in the decade since the end of rationing, they “increasingly confronted … obesity.”^47^ Like their peers elsewhere in Greater London, and like a growing body of expert opinion, school medical officers and health educators in Croydon were eager to intervene when their charges grew plump. Yet they faced resistance from parents who remembered pre-war hunger and indignantly rejected advice that children should not be “fed indiscriminately.”^48^

In this climate of growing concern about expanding (child) bodies, how was “overweight” determined by health professionals operating in UK schools? Gibbons’ 1965 report detailed a variety of means. Here, quantification certainly played a role, in the form of anthropometric surveys of schoolchildren. Their quantified weights were assessed alongside quanta of height – but interpreted by experts through the entirely qualitative category of “body build.” Comparing the results to (apparently local) means of height and weight, this work revealed that “5 - 15% of schoolchildren” were at least 10% above the mean weight for their age and body type. In the eyes of public health officials, such childhood obesity required “treatment” – and in Croydon’s schools, this prompted Gibbons to experiment with intensive and explicitly quantitative surveillance in a group setting and in the children’s homes:

As well as the regular weight recordings, the girls’ heights and girths are checked periodically; their Blood Pressures are recorded and urine tests carried out also. At the initial meeting the girls’ mothers are asked to attend as well, and the purpose and aims of the group are explained. It is stressed that the only way to reduce and control weight, is by a sensible diet which involves an overall reduction in calorie intake while maintaining an adequate protein, vitamin and mineral intake. Diet sheets have been especially prepared to achieve simplicity and fit in with the rest of the family’s meals.^49^

Clearly, this approach did not rely on weight quantification alone either to determine or to prompt action on individual obesity. Measurement was only one aspect of the intervention, which was accompanied by a wide range of educational, support, and surveillance activities designed to create an actively healthy (hygienic) citizen rather than merely a “normal” one. Recognizing that her experimental approach was complicated and time-intensive, Gibbons added, “the potential numbers needing treatment were too great to be all treated in this way”: that is, as individual idiosyncratic bodies.^50^

In 1968, even her skeptical superior acknowledged that “despite evidence of children being sent to school without breakfast, *obesity was still the greater danger to future health and longevity*,” and by 1969, Gibbons’ experimental weight control clinic had developed into borough-wide provision of school weight control clinics.^51^ Upscaling came at a cost, and with a change in focus. While nutritional education remained a popular feature of the expanded program, quantification in the form of regular weekly group weigh-ins had become the dominant intervention. Other clinical measurements taken to assess the health of affected children were apparently discarded. Weight loss, rather than health gains, were the measure of success, and the girls were assessed against the Metropolitan Life Insurance Company’s “ideal weight” charts of 1960, rather than by individual clinical scrutiny incorporating attention to body type. In this regard, such attention to *population*-based understandings of overweight foreshadows future shifts away from the individualism characteristic of British cultures of adult self-weighing.

Gibbons was not alone in expressing and acting on fears of overweight as a growing threat to child and adult health. Successive reports from concerned MOsH track levels of official concern with the rising trend in British body weights. Other local health authorities tackling overweight and obesity in this period included affluent Richmond upon Thames, where the MOH chose “Diet (obesity)” as the subject for one of its monthly poster campaigns in 1972; and Kingston upon Thames, where overweight was a persistent concern.^52^ By 1972, the economically mixed borough of Haringey had established Weight Watchers’ clinics for obese girls, and looked enviously to its neighbor in Camden and Islington, which ran holidays for similar children in 1971. It is noteworthy that as well as measuring height and weight, their service assessed obesity through “a special questionnaire — including an individual graph for each child … and, apart from check-ups of weight and height, the Blood Pressure and the thickness of the skin fold.”^53^ Here too, when professionals explored overweight in individuals, their assessments did not depend on simple height/weight ratios, but required more detailed clinical measurement. It is, of course, unsurprising that professional concerns and interventions focused first on overweight girls; as the wider literature documents, normative surveillance in relation to weight and fatness has consistently been gendered, targeting women and girls.^54^

While most MOH reports that addressed obesity in the 1960s focused on children, some foreshadowed future developments in adult health. In 1968, for example, health educators in Harrow turned their gaze to the adult male, observing that for middle-aged men in Harrow, “the percentage of total male deaths from all causes in 1966, which were due to cardio-vascular diseases was 46.25%, compared with a figure of 17.4% in 1937.” They blamed, among other factors, obesity. Like other contributory factors, it could be “controlled by the individual.”^55^ Reinforcing the implicit importance of adult personal responsibility, these health workers observed that cardiovascular disease mortality among middle-aged women, contrastingly, dropped; they compared the “diet conscious” “woman of today” favorably to her husband, “who probably pays more attention to the inner workings of his automobile than his own body.”^56^ In subsequent reports, Harrow’s MOH repeatedly and with increasing frustration located responsibility for obesity and its disease sequelae in “the individual’s jurisdiction.”^57^

This emerging push toward action on overnutrition and overweight reflected a refocusing of enduring tropes of individual moral responsibility for public health away from apparently defeated epidemic and contagious diseases toward the new chronic diseases of the day – expanding the “preventive medicine” and hygienic citizenship of the interwar years to confront new threats to personal health.^58^ This new style and focus of health promotion is exemplified in the comments of Greenwich’s MOH, J. Kerr Brown, on health education in 1965. Health education, he argued, now addressed areas “in which legislation has little or no effect”; “modern health thinking” depended on the individual “refraining from harming his or her own health.” In the absence of suitable legislative targets, Kerr Brown suggested that the deliberate inculcation of community moral opprobrium might effectively discourage such poor behavioral choices: “the aim of health education is to achieve a climate of opinion where indulgence in anti-health activities is viewed with the same distaste as infrequent bathing, spitting, etc.” Kerr Brown explicitly noted obesity as a health problem susceptible only to such persuasive and personal efforts.^59^

Unusually, in later reports Kerr Brown also hinted at almost iatrogenic origins for modern obesity, especially in children. Of course, they and their parents were susceptible to “high pressure salesmanship” in advertising; this was territory he hoped to retake through health education stigmatizing “indulgence.” However, Kerr Brown also observed that manufacturers had successfully colonized the scientific substrates of contemporary nutrition education: “Threatened with malnutrition of all kinds from avitaminosis and trace element deficiencies to a lack of energising carbohydrates if certain foods are not ingested, with minimal attention to a balanced diet, the cossetted off-spring is quickly weaned on to cereals and encouraged to over-eat by anxious, over-zealous but conditioned parents.”^60^ Facing the twinned challenges of encouraging individual moderation and the effective commercial co-option of scientific health messages, for this MOH meticulous quantification and rigorous surveillance apparently offered few obvious advantages. His reports steered clear of encouraging quantified weight surveillance. Rather, he proposed simple – but individual – practices of dietary restriction: “continue to eat the foods you like… but in only half the quantities you would normally take.”^61^

In sidelining quantification, Kerr Brown’s approach also reflected wider appreciation of a crucial problem for state actors interested in stemming the rise of obesity. At a population level, the trend of rising body weights could be tracked, at least in theory. Moreover, epidemiologists and others could suggestively link overweight to higher rates of *population* morbidity from heart disease, and later to a range of other chronic conditions. Interested hospital consultants and general practitioners too recognized the upward weight trend in their own practices (and in some case responded by writing their own diet books).^62^ However, a medical consensus on the definition and measurement of “obesity” in individual adults was proving elusive. As Kerr Brown remarked in 1971,

Use of terms such as “overweight” and “obesity” suggests the existence of a standard of normality with which comparison may be made. This is not so. Neither in this country nor any other country has really solved the problem of collating reliable information on a national scale … There is neither an ideal nor a normal weight, but only an average weight … subject to variation according to the type of skeletal frame genetically inherited.^63^

A concerned consultant similarly grumbled, “there is nothing very scientific about what we should weigh. Statistical and scientific approaches to the question of overweight become very involved and impractical. So many different opinions are expressed that confusion results.”^64^ Clinically, obesity could only be observed in and experienced by individuals, and the common sense of the postwar period asserted just as firmly as in the interwar years that only individuals could control their weight.

Whether or not the “climate of public opinion” was swayed by public health efforts to stigmatize “anti-health” indulgences, such disapproval certainly radiated from the pages of MOH reports by the 1970s. A 1971 report admonished, “[i]t is not without significance that gluttony is listed as one of the seven deadly sins for, today, we are bedevilled with freak nutritional patterns and diets which encourage the development of obesity.”^65^ Underlying such hardening attitudes was growing acceptance among public health workers and epidemiologists that being overweight was dangerous not just to the individual but to the community and country. Again, Kerr Brown put it bluntly: “obesity underlies much of the country’s ill-health” and endangered “community health.”^66^

For these professional groups, the problem was twofold. Certainly, they had to convince individual members of the public – the men and women in the street – to act on their own growing bulk, not least because of its dangers for the community in the context of a welfare state. But they had also to persuade policy makers and legislators at the national and international level that the public health threat of obesity (now regularly defined in terms of measured excesses of individual weights as compared to established weight norms for height and age), like those posed by smoking or drink-driving, required careful scrutiny, urgent action, and state intervention. In the remainder of this essay I will first briefly examine existing cultures of quantification in post-NHS personal weight management; I will then explore the ways in which the rise of a new quantitative measure, the body mass index (BMI), reframed perceptions both of obesity and of self-quantification.

## Overweight in the welfare state: self-care and the scales, 1948–79

Public self-weighing persisted and flourished in 1950s and 1960s Britain, and so did the personal scale. *The Times* newspaper assumed (perhaps prematurely) the ubiquity of such scales in the homes of their typically affluent readership as early as 1959.^67^ Bathroom scales were also a popular choice among the “luxuries or semi-luxuries” offered to smokers redeeming the gift coupons distributed with packets of cigarettes (a widespread marketing technique in 1960s Britain).^68^ Popular dieting books, too, extolled – and expected – the scales. In the 1950s, Jean Robins, a “television slimming expert,” deployed medical authority to support her advocacy of self-weighing. In the foreword to her *Common-Sense Slimming*, Dr Frank Jeffrey duly advised, “It is wise for everyone to know approximately what is her optimum weight and to weigh herself periodically.”^69^ Throughout the volume, meticulous self-weighing featured as a required and regimented part of weight loss. Robins devoted a whole section to training readers to weight themselves accurately:

One of the most important items on the programme of the reducing diet is the weekly weighing. There is no harm in weighing yourself as often as you please, but it should be done at least once a week during the dieting period… strictly according to the following rules: (1) always use the same set of scales. … Chemists’ shops and department stores are the kind of place where one expects to find really reliable scales. (2) weigh at the same time on the same day of the week. … (3) always wear the same weight of clothes … (4) keep a weight card. This is essential for your own guidance… It should record your official weekly result to the nearest ounce.^70^

Crucially, only weighing would do; Robins explicitly discarded all other means of self-assessment and weight loss as “folklore methods … picked up at school or from advertisements.” Even the measuring tape was gently mocked. And self-weighing would become a life-time discipline. Robins demanded “a regular weekly check on the same system that you used during the dieting period” to guard against weight gains. The “friendly scales” were a metric for life.^71^

By the 1960s, such careful and detailed instructions in self-weighing were no longer required, but formed part of the dieter’s assumed knowledge. In 1962, the British Medical Association’s lay health advice magazine, *Family Doctor*, merely specified “regular use of the scales, preferably in the bathroom where we can judge ourselves naked”; the article’s only additional advice was that self-weighers should consult “a table of weights and heights” to establish “a standard for our age.”^72^ A subsequent article presented the scales as “a sound investment for health.” Importantly, both articles focused specifically on voluntary and conscientious *self-*weighing by individuals intent on preserving their own health.^73^ By 1967, Marion Harris’ *The Awful Slimmer’s Book* – subtitled “Do the Scales Get Up and run?” – offered no instructions at all on how to use the scales in slimming. Across its pages, she simply referred to specific weight measurements, and relied on its readers having daily access to a personal scale as well as the “ideal weight charts” in the book’s appendix. “Your scales don’t lie,” she assured her readers, and only the scales (and explicitly not the mirror) could “tell you it’s ok.”^74^

In the 1970s, explanations of calorie counting replaced instructions on self-measurement, while calls for slimmers to seek “medical advice” returned to dieting manuals and the new “slimming” magazines. Intriguingly, it is in this decade that editors and authors of advice books begin to critique the height–weight charts that had been at the heart of quantified British self-surveillance throughout the twentieth century. In *Let’s Start to Slim*, for instance, the editors of the independent *Slimmer Magazine* observed that “charts outside the chemist’s shop can often be misleading” by failing to take stature and frame into account. They reported this as a medical concern: “one doctor specialising in weight problems illustrated the general confusion by telling me, ‘I have had patients who are obviously too fat come to me and say, “but according to the list of average weights and heights in the chemist’s shop, I’m *not* overweight,”’” and encouraged readers also to judge their weights by eye and touch.^75^ Weekly (or more frequent) self-weighing nonetheless continued as the implicit foundation of all slimming programs. Even the title page of *Let’s Start to Slim* featured a woman weighing herself on a slimline scale.^76^ In this period, too, the print press sporadically reintroduced notions of individual overweight and unfitness (sometimes visually signified by a straining or complaining scale) as an indicator of national decline or enfeeblement. These were common in the interwar period, but barely seen since 1948.^77^

By 1979, state and professional concerns about rising levels of diet-linked chronic illness prompted the establishment of the National Advisory Committee for Nutrition Education, while wider economic retrenchment and political changes favoring markets and individual consumerism drove a reconsideration and re-evaluation of preventive medicine as a cost-saving device for the hard-hit NHS.^78^ This conjunction of trends would have profound effects on popular discourses of weight management and obesity.

Looking beyond the advice literature and into British homes to gauge the uptake of daily or regular self-weighing is harder. However, a 1967–8 Mass Observation Ltd. Study offers a rare glimpse of domestic practices among British women seeking to manage their weight, and that of their families. The study, performed by “food consultant” and nutritionist J. C. McKenzie, was based on qualitative observations of 52 women, evenly split between self-describedly “successful” and unsuccessful slimmers, and a survey of a nationally representative sample of 2,000 adults, in May 1968. This work confirmed that the researchers and most participants took self-weighing for granted as integral to domestic practices of weight management and assessments of its success. While the precise measurement of food items and physical dimensions such as hip and arm circumference attracted explicit attention, exact weight measurements – fundamental to much of the reported testimony from individual slimmers – appeared without explanation: “When I get to 10 stone I diet to 9 stone 4 lbs. and then I do a day a week to keep it that way,” recalled one woman. For the researchers, such “precisely defined” and specifically quantified goals are the identifying feature of “Successful Slimmers.”^79^

Another group noted by the author as successful in weight management were the “Weight Watchers.” As well as avoiding “fattening foods” for themselves and their families, they too both self-reported assiduous scale use, and were observed to be committed to both self- and family surveillance: “I watch my weight all the time. I try to keep to the right weight for my age and size.” Even “Unsuccessful Slimmers” with long-term weight problems deployed the language of quantified weight: “I ought to lose 2 or 3 stone.” However, the researchers reported that they were far less precise in their goals, and spoke more about experiential cues, like “*figure deterioration*.”^80^ Precision in both measurement and aspiration, then, were naturalized as keys to weight-management success.

The study’s observations and the results of the survey mirror representations of overweight in the press. The researchers also spotted the effects of increased health reporting and public health messaging linking male obesity to heart disease.^81^ While for women themselves, “the health factor” paled in comparison to “the feeling that society caters more effectively for the relatively slim person” and regarded slenderness as attractive, in relation to their husbands, “the issue is very different”: “They seem less concerned with the aesthetic picture … but they were concerned about the effect upon his health. They felt he should get rid of weight because he was jeopardising his health.”^82^ These parallels between media discourse and domestic practice are unsurprising: in a 1967 survey of a “national quota sample” of 2,000 individuals, large numbers reported taking their weight-management advice from articles and advertisements in the newspapers and magazines. From observation and survey data, the researchers concluded that “weight reduction is widely discussed between friends and relations, and that the papers are carefully scrutinised for information on this subject.”^83^

The scale, and by implication the individually enacted practice of self-weighing, was an enduring feature of the myriad popular accounts of personal overweight and its management in the period between 1948 and 1979. Scales might in this period be faced by their users with resignation, trepidation, or even indignation; they might chastise or reward; but they remained emblematic of chosen, rather than imposed, individual regimes of weight loss as self-care. At least in the popular discourse of postwar Britain, fat was a personal and not a political issue. State interventions in this culture of self-weighing, whether active or advisory, were rarely welcomed, or even taken seriously.^84^ Professional discourse observed and cautioned against overweight and dietary indulgence (and indeed often condemned the British diet wholesale), but positioned obesity as the result of misguided or misinformed individual or parental choices. Dismissing top-down interventions, doctors and others encouraged individuals to adopt a moralized pattern of self-control, operated and assessed specifically through the familiar task of domestic self-weighing. The NHS was almost invisible in obesity discourse during this period; early optimism in curative therapies for overweight was tarnished by iatrogenic addiction crises, while even the “new public health” was ill-prepared to tackle lifestyle diseases prompted by something as essential as food, and as personal as dietary choice.^85^ The popular press meanwhile alternately ridiculed and humorously commiserated with the overweight.

However, from the 1970s onwards, popular discourses of overweight turned deadly serious, and in succeeding decades, obesity and the ways, spaces, and cultural context in which it was measured changed radically.^86^ Today, professionals clamor for top-down interventions like the recent “sugar tax,” while popular discourse predicts disaster for overweight individuals and the NHS alike. Weight is once again a matter of state. To understand this radical shift, and to explore the changing tone of British obesity discourse after 1980, it is worth looking at the rise and rise of the BMI.

## “Simple” measures and epidemic predictions: the uses of the BMI

Scholars have noted critically the growing state and professional consensus supporting “simple” health advice, health promotion techniques, and health education messages in the twentieth century.^87^ Hewing very closely to this line, early publicity and health campaigns around Britain’s new NHS stressed the “simple” “Seven Rules of Health,” and similarly straightforward, quanta-free messages related to diet and nutrition.^88^ Yet in relation to complex conditions such as overweight and obesity, what work does the “simpleness” of “simple” rules and “simple” measurements do? In part, it erases the complexity and contingency of arguments about lifestyle or behavioral “risks” that have dominated public health and epidemiological thinking about chronic conditions since the 1960s. Claims rooted in statistical and population studies are thus converted into health education messages that target individuals and can be operationalized through screening, mass media, and marketing campaigns, even in the absence of professional consensus.^89^

The body mass index offers a clear demonstration of this process. BMI was a tool originally conceived by nineteenth-century statistician and widely acclaimed progeniture of the “average man,” Adolphe Quetelet; it was first widely used by actuaries for major life insurance companies at the beginning of the twentieth century.^90^ An individual’s BMI is calculated by dividing the body mass (weight in kilograms) by the square of body height (in meters). From the mid-century, BMI was used by epidemiologists, public health workers, and anthropometrists as a proxy indicator of healthy weight, despite its well-rehearsed limitations (for instance, BMI is unable to account for the greater weight of muscle than of fatty tissue, or for the differential risks imposed by varying patterns of fat distribution). Isabel Fletcher has made a compelling case that the adoption and promotion of BMI as a “simple numerical index” of obesity made it possible for researchers and policy makers to claim that the rise in average body weights in U.S. and UK populations was “an important health problem,” even an “epidemic.” BMI data could also be dramatically visualized using tropes already familiar to expert and lay audiences alike from representations of past epidemics.^91^ Its “simplicity” – both of production, since determining BMI required only a measuring tape, a weighing machine, and one calculation; and of comparison, as a simple numerical absolute measure – also featured strongly in Ancel Keys’ 1972 paper, which established BMI as the “gold standard” measurement for obesity, and has remained a central claim for its global users and popularizers ever since.^92^

Yet as many researchers have discussed, and as expert proponents of BMI from Quetelet to Keys and beyond acknowledged, BMI was developed to enable expert anthropometric and epidemiological comparisons between populations, not as a clinical tool for assessing individual health, and still less as a useful quantum of health self-knowledge.^93^ In Britain, BMI remained a term of art, used almost exclusively by experts until the late 1980s. Where and when did BMI enter popular discourse, and how did this “simple measure” contribute to the sharp change in tone of newspaper coverage of obesity after the 1980s? In the final section, I will explore the (re)birth of healthy weight as a marker of civic responsibility and hygienic citizenship in the era of obesity as a threat to the NHS.

The British press showed little initial enthusiasm for BMI. As we have seen, public health workers working on the front line with individual members of the public also turned only reluctantly and under the rising pressure of numbers to the exclusive use of weight and height data as the markers of obesity. It was this rising volume of cases, along with growing expert and policy attention to the theorized role of excess weight as a risk factor in chronic diseases (first coronary heart disease and then non-insulin dependent diabetes), that provoked a gradual shift in the tone and content of news coverage of obesity. And even this potent combination might not have been enough to strip overweight of its individualized and often humorous connotations, without the complicating factor that the increased medicalization of overweight – promoted both by epidemiology and by new treatment modalities – piled increasing pressure on the perennially “cash-starved” NHS.^94^

The first signs of this shift emerge in the mid and late 1970s, as public attention focused on “slimming drugs,” their risks, and especially their cost to the NHS. Official admonitions urging general practitioners (GPs) to reduce their spending on slimming drugs in 1976 reflected a wider re-moralization of the issue of overweight in a period when the NHS and the nation faced significant economic challenges. Not everyone agreed. Older models of overweight as the results of psychosocial factors, alongside new recognition of powerful commercial interests at play in the matter of dietary choice, persuaded some that NHS intervention remained worthwhile. As one medic pleaded, “Of course the Minister for Health, Dr David Owen is correct; the application of willpower is a better slimming aid than appetite suppressant drugs supplied at a cost of £2.5 million a year through the NHS” – but without them and “the associated regular morale boosting visits to the doctor and the corner chemist,” the dieter was doomed to exploitation by the “diet industry.”^95^

Such pleas notwithstanding, governments in the UK remained reluctant to intervene against obesity forcefully either through regulation or taxation.^96^ Notably, understandings of the government’s role in promoting and protecting “public health” had changed substantially since the regulatory heights of rationing. As one influential nutrition worker in the Department of Health and Social Services (DHSS) summarized in 1977, “[n]utritional problems can be dealt with either by changes in national policy or locally by area health authorities. Alterations in national policy are in general reserved for problems which affect the national health and which can only be solved by Government action.”^97^ Adult overweight and obesity, long linked to individual choices, and apparently producing “only” individual risks, did not (yet) meet this high standard. A culture in which weight assessment was a matter for individual self-measurement, whether in the privacy of the domestic bathroom or the voluntary public weigh-ins of the slimming club, reinforced this perspective. Moreover, like governments around the world, the British state still had little appetite for action in the interests of public health against the established interests of the food and diet industries.^98^

Others argued that the NHS could not provide the “individual treatment” required to medically encourage and sustain weight loss, and that it was “unrealistic to expect the NHS to treat all overweight patients.” Dieters should instead pay to join commercial slimming clubs, where “authoritarian” rules and public weighing would stiffen their will.^99^ Conservatives even argued that “the NHS should charge for treating what, in effect, are self-inflicted illnesses … like non-glandular obesity.”^100^ The need to educate the public to regulate themselves became political and policy common ground, with many citing the success of health education campaigns in reducing smoking and drink-driving (while ignoring the importance of taxation and legislation, respectively, in those phenomena). “Action,” as Sylvia Darke put it on behalf of the DHSS, “must be based on sound evidence and on sound nutrition education.”^101^

For some in the press and the health professions, quantification per se looked like a useful solution to what they perceived as public confusion about “the links between food and health”; as one article in *The Guardian* asked, “How many greasy chips constitute a health hazard?” Reporting “disgust” among community health educators at the role played by commercial bodies in public nutrition education, they and the article demanded the translation of “scientific dietary goals into practical advice” through, specifically, quantified dietary guidelines modeled on those in the United States and Scandinavia.^102^ The *Financial Times*, too, called for (NHS funded, and clinical) measurements as a tool of prevention and an aid to personal responsibility, and critiqued Britain for its failure to emulate the United States and Australia by making a “national effort to lower risk factors and improve lifestyle.”^103^

By 1988, a clear discourse relating population health to national status has re-emerged in the national press, this time in relation not to malnutrition or infant and maternal welfare as in the first half of the century, but to the chronic “lifestyle” diseases.^104^ Responding to the government’s 1987 primary health White Paper, *Promoting Better Health*, the *Financial Times* was particularly blunt: “the UK is being described as the Sick Man of Europe because it has begun to lag behind most other developed countries in preventing disease and promoting good health.” The NHS, its reporter Alan Pike suggested, had been distracted from its “founding aims” of promoting health and preventing illness by the “dramatic and costly activities” of curing the sick.^105^ However, “solutions” to the high costs of ill-health remained “in the hands of individuals,” albeit implicitly well-informed and rational ones. Associations between declining national standing and soaring national bodyweights would continue through the 1990s and into the twenty-first century.^106^

Surprisingly, given its ubiquity in the professional literature, the earliest national press coverage deploying BMI as a health indicator that I uncovered was a critical piece published in the left-leaning *Guardian* in 1987. In “Fat is a Positional Issue,” nutrition researcher Michael Gibney introduced his readership to the BMI and its appeal. “Measuring human body fat isn’t easy,” he observed, describing the variously specialist, uncomfortable, and invasive techniques required to accurately assess individual body fat. BMI was the “least invasive,” and as only “[l]arge-scale studies” could identify causal factors in chronic disease, BMI had become the “favoured” method of those eager to explain and quell the rise in heart disease, diabetes, and chronic conditions of affluence. Gibney strongly disputed the value of BMI for predicting coronary heart disease, observing that the ratio of waist:hip circumference (WHC), in contrast, was a “powerful predictor.” This complaint reflected abiding professional doubts over the value of BMI as a metric of individual health – but the WHC never gained equal standing with the apparently more scientific (and as we will see, state-privileged) BMI. As one reporter observed wryly, while doctors were enthused by the predictive value of the WHC for coronary and other chronic diseases, “many gave it up after seeing [patients’] looks of amazement … when their medical advisers suddenly produced a tape measure and said that their next test was to have their bottom measured.”^107^ Here, the very simplicity of a “simple measurement” discouraged its adoption. BMI, consistently accompanied in early press coverage by equations and often charts to assist the reader, was clearly just complicated enough to seem “scientific.”

In 1989, *The Times* printed a reader’s letter addressing the BMI metric that sheds useful light on its increasing visibility. The author, herself a GP, offered an amused commentary on the new GP contract’s stipulation that she should measure the heights and weights of all her patients between 16 and 74 years of age triennially. While she could “hope to influence their lifestyle” to encourage attainment of “a desirable body mass index,” she observed mordantly that “no amount of exhortation on my part will induce any of them to change their height.” The correspondent, Elizabeth Ruttley, did not mention that for taking each of these measurements, she and her fellow GPs were to be rewarded by additional fees as part of a new cost-cutting drive for “preventive” NHS care.^108^ As Williams et al. observe, this marketization of preventive health measures, and the focus on the quantitative assessment of individuals’ health, all fit well with the then-prevalent government interest in target-driven managerialism, small-state economic efficiencies, and ideological promotion of “self-reliance and individual responsibility in all walks of life, including health.”^109^ Driven by this state agenda, for the NHS quantification became ostensibly synonymous with “prevention,” despite the obvious gap between numerical measurements and clinical outcomes, and between individual self-knowledge and active self-care through, for example, weight loss or dietary reform.

Once BMI was thus firmly embedded as a staple of NHS provision and health education initiatives, it appeared regularly in the national papers. The term “body mass index” featured in 109 articles in *The Times* between 1989 and 2004; 101 pieces in *The Guardian* between 1987 and 2004; and another 144 in the mass-market national tabloid the *Daily Mail* between its (belated) first appearance in 1990 and 2004.^110^ The *Daily Mail* routinely described BMI as “the most accurate way of assessing your weight and shape,” while *The Times* and *The Guardian* were more likely to simply assume the metric.^111^ Interestingly, despite their extensive discussion and use of BMI, and despite its position as the “official” metric of overweight, reporting in all three of these national news outlets intermittently questioned its value and the value of weight quantification as a measure of health status. For instance, Muir Grey, then-Director for the UK National Screening Programme, was scathing about the stress on measuring BMI in 1999, advising readers: “You’d be better off taking your clothes off, looking in the mirror and being honest.”^112^ Another article (representative of a minor theme across the papers) complained that, in BMI terms, international rugby star “Jonah Lomu is fat.” Reporter Michael Hann pointed out that “in individual cases the formula is not as helpful as you might believe. … The simplicity of the BMI makes it a godsend for looking at trends, but it is also something of a broad-brush tool,” unable to account for the location of body fat, the greater density of muscle, or different healthy levels of body fat across age, gender, and “racial” groups.^113^

Nonetheless, by the 1990s, coverage of overweight was consistently framed in terms of (quantified) obesity and BMI. The emotional register of such articles ranged from serious to near-hysteria. Here too, the role of changes in, and pressures on, the NHS are prominent. By 1993, *The Guardian* ran an obesity story under the headline “Living off the Fat of the Land.” The article was serious in tone, and noted both the lack of NHS resources for weight loss and perceptions that “the notorious side effects of the amphetamines have blown away the reputation of drug therapy as a credible aid to slimming and reinforced the view that obesity is greed to be punished, not sickness to be cured.” Here, as elsewhere, quantified self-surveillance did double duty as therapy and sanction.

In this period too, the press begins to reflect ideas of obesity as a threat to the NHS. The language of “cost” – also, of course, a quantifiable measure – begins to appear in the headlines as well as the body text. One short *Guardian* piece, covering a report from the Office of Health Economics (OHE) in 1994, asserted that “Obese people are costing the National Health Service some £200 million a year and shortening their lives, says a report out today”; the terms “cost” and “costing” appear nine times. The *Daily Mail* also reported the OHE’s conclusions under the attention-grabbing headline “£200m Bill for the Fat of the Land.” Repeatedly emphasizing the cost of treatment for obesity and obesity-related illness to the NHS, the paper also observed that in the eyes of the OHE, obesity was “easily preventable.”^114^

From this point, the return to discourses of weight and dietary self-management as “national duty” last seen in the 1950s (and last prominent in the 1930s) was perhaps inevitable. Across the 1990s and into the 2000s, this rhetoric became ever more visible. In 1993, for example, *The Independent* cited a Labour Party Conference proposal to impose “new contracts to force patients to acknowledge their responsibilities for their own heath” and “recognise the duty they owe” to the NHS.^115^ By 1998, the *Daily* Mail howled that “One Briton in two is warned over weight.” The article cited an unpublished report claiming that “health problems caused by overweight cost the NHS £1million a day.” A year later, the paper’s estimate of the bill had grown to “£1.7bn” a year – and still worse, the paper groaned, “we even outweigh the Germans.”^116^ In 2001, “Why being obese is bad for the country” was front page headline material in *The Guardian*: “We are changing shape, our health is suffering and it is costing the country a fortune … the National Health Service bill for treating the problems caused by excess weight may run to billions.”^117^ Talk of an obesity “epidemic” permeated every paper’s coverage, and added to the intensity with which the overweight were condemned as “lazy” or gluttonous.^118^ Such claims were driven by the use of BMI not just to assess and predict UK levels of obesity, but to compare the nation to others, and in particular the United States.^119^ If, in 1947, citizens were instructed by scale manufacturers to “check your weight daily” as part of the “National Duty to keep fit,” in the 2000s, beleaguered Britons were prodded: “So how do you measure up?”, before facing instruction in how to reduce their sloth, fight their gluttony, and calculate their own BMI (or occasionally another metric).^120^

## Conclusions

In 2004, a barrage of consultations and reports addressing obesity appeared in quick succession, emanating from the Houses of Parliament, the Treasury, the Department of Health, and independent think-tanks. They painted a depressing picture. The parliamentary Health Select Committee in particular envisioned a dystopic future of obesity-linked amputations, blindness, organ failures, and shortened lives. Britain’s “big-food, little-effort lifestyle” was the problem, but with whom lay the blame? For the World Health Organization’s director of chronic disease prevention, it lay with the government, which had failed to set “the conditions which allow individuals to make healthy choices.”^121^ Others blamed the public, some of whom “do not recognize obesity.” In November 2004, the UK government published a policy document called “Choosing Health: Making Health Choices Easier,” based on a major public consultation done earlier in the year.^122^ Having in previous years tested public and press responses to widely trailed proposals of more active interventions, and with no more appetite for regulating industry than the preceding Conservative administrations, “Choosing Health” was New Labour’s response to what policy makers, professionals, and journalists now routinely portrayed as an “epidemic” of obesity in Britain. Citing both rising media attention to obesity and a series of Select Committee and Treasury reports exploring the resource needs of the future NHS, the document rejected what it portrayed as polarized options: either a “paternalistic state” limiting choice and banning unhealthy behaviors or a permissive and largely absent one, leaving health to the individual and the market. Forewords by Tony Blair and Health Minister John Reid echoed uncannily the queasy ambivalence of the interwar British state toward state-sponsored “health” and fitness interventions: “Government cannot – and should not – pretend it can ‘make’ the population healthy … it is for people to make the healthy choice if they wish to. *Choosing health* sets out what this Government will do the help them.”^123^ Yet at the same time, “the improvement of everyone’s health” was “everyone’s concern” and “the Government cannot simply leave it up to individuals” – hinting at some sort of public/private panopticon.^124^

Crucially, this response demonstrated the persistence with which obesity was understood to be rooted in private “responsibility” and “individual” choices, even as successive British Attitudes Surveys from 1983–2004 indicated that the British public consistently placed responsibility for health *in general* at the door of the state.^125^ As the newspaper coverage discussed here has indicated, while the growing sense of crisis that surrounded overweight certainly shifted the valence of “fat” from humorous to horrifying, it did not generate substantial enthusiasm for state imposed dietary controls. Almost no one demanded a return to the National Loaf or butter rationing. The press, particularly the center-right *Times*, complained as frequently about the provision of obesity treatments (whether pharmaceutical or surgical) on the NHS as they did about “non-stop nannying” efforts by successive administrations seeking to persuade the nation to eat more healthily. Indeed, the intense gloom of official pronouncements in 2004 prompted resistance in some sectors of the press. The same “anti-nannying” *Times* editorial rebuked the hyperbolic rhetoric and epidemic imagery.^126^

And yet, representations of obesity as an epidemic, enabled by the naturalization of BMI as a simple diagnostic (and prognostic) tool applicable to individuals as well as populations, had produced some changes. Self-quantification played a central role in individual weight management across the lifespan of the NHS. However, until the late 1980s such efforts were, and were represented as, rightfully private and personal activities, in which professionals and the public alike interpreted absolute quantitative weight (and height) measurements alongside experiential cues and aspects of individual embodiment. “Ideal weight” charts and similar comparative tools had a place in these practices, but their variability and familiar limitations left room for individual interpretation. Weight and self-weighing tapped into and reinforced a long-established discourse of the self, linking health, appearance, behavior, and morality – but were operationalized strictly at the level of individual bodies, by individual choice. BMI, despite using almost exactly the same measurements to quantify the individual, contrastingly spoke to a discourse strictly of relative health, and implicitly configured and assessed its human objects in relation to an abstract population. Moreover, in contrast to the bathroom scale, the use of BMI was not gradually adopted by individuals, but was visibly and rather swiftly imposed, top-down, on practitioners and their patients by a worried and cost-conscious state. With the rise of BMI as the UK (and indeed international) official metric of overweight, the problem of overweight, too, was transformed from one affecting individuals to one affecting society and nation. And while this was not unique to Britain, talk of an “obesity epidemic” gained rhetorical and political traction in the UK from its predicted implications for the entire nation via its effects on the NHS. Did the provision of universal healthcare funded from general taxation therefore change British discourse about obesity? Certainly – but not immediately. Only when BMI facilitated the re-reconfiguration of individual overweight as a burden on, and thus a risk to, others – through the logic of obese bodies’ overwhelming (but “self-inflicted”) “costs” to the NHS – could overweight become, like smoking and drink-driving, an acceptable target for active state rebuke and intervention.

## Footnotes

1. E.g. Philip S. Cho, Nathan Bullock, and Dionna Ali, “The Bioinformatic Basis of Pan-Asianism,” *East Asian Science, Technology and Society: An International Journal* 7(2) (2013): 283–309; Kate Crawford, Jessa Lingel, and Tero Karppi, “Our Metrics, Ourselves: A Hundred Years of Self-Tracking from the Weight Scale to the Wrist Wearable,” *European Journal of Cultural Studies* 18(4–5) (2015): 479–96; Ulfried Reichardt, “Counting Success and Measuring Value: Money, Numbers, and Abstraction in Theodore Dreiser’s *Sister Carrie*,” *Studies in American Naturalism* 12(1) (2017): 89–104.

2. Reichardt, “Counting Success,” 91–4 (note 1).

3. James Vernon, *Hunger: A Modern History* (London: Belknap Press of Harvard University Press, 2007), pp.210–35, speaks to the cultural impacts of rationing; on the introduction of precision measurement into domestic life, see e.g. Rima D. Apple, *Mothers and Medicine: A Social History of Infant Feeding, 1890–1950* (Madison: University of Wisconsin Press, 1987); Rima D. Apple, “Constructing Mothers: Scientific Motherhood in the Nineteenth and Twentieth Centuries,” *Social History of Medicine* 8(2) (1995): 161–78; Amy Sue Bix, “‘Equipped for Life: Gendered Technical Training and Consumerism in Home Economics, 1920-1980,” *Technology and Culture* 43(4) (2002): 728–54; Lyubov G. Gurjeva, “Child Health, Commerce and Family Values: The Domestic Production of the Middle Class in Late-Nineteenth and Early-Twentieth Century Britain,” in Marijke Gijswijt-Hofstra and Hilary Marland (eds.), *Cultures of Child Health in Britain and the Netherlands in the Twentieth Century* (Amsterdam: Rodopi, 2003), pp.103–25; Lawrence T. Weaver, “In the Balance: Weighing Babies and the Birth of the Infant Welfare Clinic,” *Bulletin of the History of Medicine* 84(1) (2010): 30–57; Caroline Lieffers, “‘The Present Time is Eminently Scientific’: The Science of Cookery in Nineteenth-Century Britain,” *Journal of Social History* 45(4) (2012): 936–59.

4. This literature is too expansive for comprehensive citation, but in relation to body weight, includes: Keith Walden, “The Road to Fat City: An Interpretation of the Development of Weight Conciousness in Western Society,” *Historical Reflections/Réflexions Historiques* 12(3) (1985): 331–73; Hillel Schwartz, *Never Satisfied: A Cultural History of Diets, Fantasies, and Fat* (New York: Free Press, 1986); Peter N. Stearns, *Fat History: Bodies and Beauty in the Modern West* (New York: New York University Press, 1997); Kerry Segrave, *Obesity in America, 1850-1939: A Social History of Social Attitudes and Treatment* (Jefferson, NC: McFarland, 2008); Deborah I. Levine, “Managing American Bodies: Diet, Nutrition, and Obesity in America, 1840-1920,” unpublished PhD dissertation, Harvard University, 2008; Amy Erdman Farrell, *Fat Shame: Stigma and the Fat Body in American Culture* (New York: New York University Press, 2011); Charlotte Biltekoff, *Eating Right in America: The Cultural Politics of Food and Health* (Durham, NC: Duke University Press, 2013).

5. Tom Crook, “Sanitary Inspection and the Public Sphere in Late Victorian and Edwardian Britain: A Case Study in Liberal Governance,” *Social History* 32(4) (2007): 369–93; Tom Crook, *Governing Systems: Modernity and the Making of Public Health in England, 1830–1910* (Oakland, California: University of California Press, 2016), pp.245–86; Roberta Bivins and Hilary Marland, “Weighting for Health: Management, Measurement and Self-surveillance in the Modern Household,” *Social History of Medicine* 29(4) (2016): 757–80.

6. E.g. John Hutchinson, *The Spirometer, the Stethoscope, & Scale-Balance; Their Use in Discriminating Diseases of the Chest, and Their Value in Life Offices; With Remarks on the Selection of Lives for Life Assurance Companies* (London: John Churchill, 1852), p.359; “Collective Investigation of Disease,” *British Medical Journal* (*BMJ*), November 3, 1883, 891–2, at 891; Bivins and Marland, “Weighting for Health” (note 5). For the ambivalence of physicians towards the value of weight measurement and standardized height and weight tables as diagnostic tools in individual health, see Annemarie Jutel, “The Emergence of Overweight as a Disease Entity: Measuring Up Normality,” *Social Science & Medicine* 63 (2006): 2268–76, 2270–71.

7. Martin Powell, “Neo-Republican Citizenship and the British National Health Service Since 1979,” in Frank Huisman and Harry Oosterhuis (eds.), *Health and Citizenship: Political Cultures of Health in Modern Europe* (London: Pickering & Chatto Publishers, 2014), pp.177–90.

8. See Joanna Bourke, *Dismembering the Male: Men’s Bodies, Britain and the Great War* (Chicago: Chicago University Press, 1996); Ina Zweiniger-Bargielowska, “The Culture of the Abdomen: Obesity and Reducing in Britain, circa 1900–1939,” *Journal of British Studies* 44(2) (2005): 239–73; Ina Zweiniger-Bargielowska, *Managing the Body: Beauty, Health, and Fitness in Britain, 1880-1939* (Oxford: Oxford University Press, 2010).

9. George Orwell, *The Road to Wigan Pier* (London, 1936), pp.179–81, cited in Zweiniger-Bargielowska, “Culture of the Abdomen,” 243 (note 8).

10. Zweiniger-Bargielowska, *Managing the Body* (note 8); Charlotte Macdonald, *Strong, Beautiful, and Modern: National Fitness in Britain, New Zealand, Australia and Canada, 1935-1960* (Vancouver: UBC Press, 2013).

11. For details of the campaign, Macdonald, *Strong, Beautiful, and Modern*, pp. 35–69, esp. 51–7 (note 10); Zweiniger-Bargielowska, *Managing the Body*, p.309 (note 8).

12. Quoted in Macdonald, *Strong, Beautiful, and Modern*, p.56 (note 10). See also Jane Seymour, “Not Rights but Reciprocal Responsibility: The Rhetoric of State Health Provision in Early Twentieth Century Britain,” in Alex Mold and David Reubi (eds.), *Assembling Health Rights in Global Context: Genealogies and Anthropologies* (Abingdon: Routledge, 2013), pp.23–41.

13. Quoted in Zweiniger-Bargielowska, *Managing the Body*, p.319 (note 8).

14. For Britain, see Bivins and Marland, “Weighting for Health” (note 5); Macdonald, *Strong, Beautiful, and Modern* (note 10); Vernon, *Hunger* (note 3); Zweineger-Bargielowska, “Culture of the Abdomen” (note 8); Zweineger-Bargielowska, *Managing the Body* (note 8).

15. “The Nation’s Health,” *The Times*, February 5, 1937.

16. Zweiniger-Bargielowska, *Managing the Body*, pp. 151–92 (note 8); Vernon, *Hunger* (note 3).

17. Zweiniger-Bargielowska, “Culture of the Abdomen,” 272 (note 8); Bivins and Marland, “Weighting for Health,” 776 (note 5).

18. Alex Mold, “Exhibiting Good Health: Public Health Exhibitions in London, 1948-71,” *Medical History* 62(1) (2018): 1–26, 15.

19. Jutel, “Measuring Up Normality,” 2270–2 (note 6).

20. E.g. H. M. Sinclair, “Assessment and Results of Obesity,” *BMJ*, December 26, 1953, 1404–6.

21. Avner Offer, “Body Weight and Self-Control in the United States and Britain since the 1950s,” *Social History of Medicine* 14(1) (2001): 79–106; Zweiniger-Bargielowska, “Culture of the Abdomen” (note 8).

22. “Old English Custom,” *The* *Times*, August 16, 1956.

23. “Old English Custom,” *The Times* (note 22).

24. Mark Bufton, David F. Smith, and Virginia Berridge, “Professional Ambitions, Political Inclinations, and Protein Problems: Conflict and Compromise in the BMA Nutrition Committee 1947-1950,” *Medical History* 47 (2003): 473–92, 473–6.

25. Roberta Bivins, “Ideology and Disease Identity: The Politics of Rickets, 1929–1982,” *Medical Humanities* 40 (2014): 3–10; Anne Murcott, “Food and Nutrition in Post-war Britain,” in Peter Catterall and James Obelkevich (eds.), *Understanding Post-War British Society* (London: Routledge, 1994), pp.155–64, 157–9; Vernon, *Hunger*, 159–95, 196–235 (note 3); Charles Webster, “Government Policy on School Meals and Welfare Foods, 1939-1970,” in David F. Smith (ed.), *Nutrition in Britain: Science, Scientists and Politics in the Twentieth Century* (London: Routledge, 1997), pp.190–213. In this they reflected continuities rather than radical change: Jane Lewis, *The Politics of Motherhood: Child and Maternal Welfare in England, 1900–39* (London: Croom Helm, 1980); Celia Davies, “The Health Visitor as Mother’s Friend: A Woman’s Place in Public Health, 1900–14,” *Social History of Medicine* 1(1) (1988): 39–59; also Mold, “Exhibiting Good Health,” 15 (note 18).

26. E.g. Mold, “Exhibiting Good Health” (note 18).

27. Wellcome Library (London), 811058i “The ‘Health of the People’ Exhibition, Oxford Street, London,” 1948.

28. Ibid., p.4.

29. Ibid., photograph album, image D 39477.

30. Here I used the Wellcome Library’s “London’s Pulse: Medical Officer of Health Reports 1848-1972” <https://wellcomelibrary.org/moh/> (19 February 2018) (hereafter, “London’s Pulse”), searching all Reports between 1948 and 1972. While these reports reflect conditions only in the Greater London metropolis, they therefore include districts across the economic spectrum from extreme deprivation to extreme wealth.

31. London’s Pulse, search term: “obesity”; search parameters: all areas, between 1948 and 1972, identifying 153 individual reports.

32. London’s Pulse, Andrew M. Forrest, “Borough of Leyton Health Report for the Year 1951,” p.102.

33. London’s Pulse, A .T. W. Powell, “Report of the Medical Officer of Health for Walthamstow, 1954,” pp.72–3.

34. London’s Pulse, S. L. Wright, *Annual Report of the Medical Officer of Health and Principal School Medical Officer, 1956*, Appendix B, p.7.

35. Ibid., p.7.

36. Mark Bufton, “British Expert Advice on Diet and Heart Disease,” in Virginia Berridge (ed.), *Making Health Policy: Networks in Research and Policy after 1945* (Amsterdam: Rodopi, 2005), pp.125–48, 131. The Committee in question was the Standing Committee on Medical and Nutritional Problems from 1941–1957, then the Committee on Medical and Nutritional Aspects of Food Policy, or COMA.

37. London’s Pulse, John Maddison, *Good Health in Twickenham, 1952*, p.10.

38. Ibid., p.7.

39. Ibid., p.8.

40. Ibid., p.8.

41. Ibid., p.7.

42. Ibid., p.9.

43. This was already a shift from the top-down, perhaps rather passive health “rights” of Marshallian citizenship described in Powell, “Neo-Republican Citizenship,” pp.178–9 (note 7).

44. Macdonald, *Strong, Beautiful, and Modern*, p.152 (note 10).

45. Increasing awareness of links between a fatty diet and adult coronary heart disease drove this change; see Mark Bufton and Virginia Berridge, “Post-war Nutrition Science and Policy Making in Britain c. 1945-1994: The Case of Diet and Heart Disease,” in David F. Smith and Jim Phillips (eds.), *Food Science Policy and Regulation in the Twentieth Century: International and Comparative Perspectives* (London: Routledge, 2000), pp.207–22.

46. London’s Pulse, S. L. Wright, *Borough of Croydon Annual Report of the Principal School Medical Officer of Health for Croydon, 1957*, p.7.

47. London’s Pulse, S. L. Wright, *Borough of Croydon Annual Report of the Principal School Medical Officer of Health for Croydon, 1965*, “APPENDIX E: An Approach to the Treatment of Overweight Adolescents by Phyllis M. Gibbons (School Medical Officer),” pp.64–6.

48. Wright, *Annual Report of the Principal School Medical Officer, 1965*, p.18 (note 47).

49. London’s Pulse, Gibbons, “Overweight Adolescents,” pp.64–6 (note 47).

50. Ibid., pp.67–8.

51. London’s Pulse, S. L. Wright, *Borough of Croydon Annual Report of the Principal School Medical Officer of Health for Croydon*, 1968, p.2; and in the same volume, Phyllis M. Mortimer, School Medical Officer, “Weight Control Clinic,” pp.31–3.

52. London’s Pulse, A. M. Nelson, *London Borough of Richmond Upon Thames Annual Report of the Medical Officer of Health and Principal School Medical Officer for the year 1972*, p.34.

53. London’s Pulse, J. L. Patton, *Haringey Health in 1972*, p.77.

54. See notes 1 and 4.

55. London’s Pulse, William Cormack, *London Borough of Harrow Annual Report of the Medical Officer of Health and Principal School Medical Officer for the year 1968*, p.51.

56. Ibid., p.51.

57. London’s Pulse, William Cormack, *London Borough of Harrow Annual Report of the Medical Officer of Health and Principal School Medical Officer for the year 1969*, p.17.

58. E.g. Jane K. Seymour, “Not Rights but Reciprocal Responsibility,” pp.23–41, 38–9 (note 12).

59. London’s Pulse, J. Kerr Brown, *Report of the Medical Officer of Health for Greenwich Borough, 1965*, p.264.

60. London’s Pulse, J. Kerr Brown, *Report of the Medical Officer of Health for Greenwich Borough, 1969*, p.68. See also Jane Hand, “Marketing Health Education: Advertising Margarine and Visualising Health in Britain from 1964–c.2000,” *Contemporary British History* 31(4) (2017): 477–500.

61. Kerr Brown, *Report, 1969*, p.69 (note 60).

62. E.g. Stephen Taylor, *Fats and Figures: Slimming Without Fears* (London: Andre Deutsch, 1951); Dennis Craddock, *Obesity and its Management* (London: E.S. Livingstone, Ltd, 1969); Robert Kemp, *Nobody Need Be Fat* (London: William Heineman, 1959).

63. London’s Pulse, J. Kerr Brown, *Report of the Medical Officer of Health for Greenwich Borough, 1971*, pp.92–3.

64. Kemp, *Nobody Need Be Fat*, p.14 (note 62).

65. Kerr Brown, *Report*, 1971, p.90 (note 63).

66. Ibid., p.90.

67. Peta Fordham, “All Ways at Home,” *The Times*, April 27, 1959.

68. “Britain’s Changing Society: More Gift Coupons with the Puffs,” *The Times*, May 11, 1966.

69. Jean Robins, *Common-Sense Slimming* (London: Odhams Press, n.d. but no later than 1954), p.7.

70. Ibid., pp.32–3.

71. Ibid., p.63.

72. Harvey Williams, “Is Dieting Worth While?” *Family Doctor* 12 (2) (1962): 94–5, 95.

73. Dr Kenneth C. Hutchin, “Stop Killing Yourself with Kindness,” *Family Doctor* 12(12) (1962): 742–44, 742.

74. Marion Harris, *The Awful Slimmer*’*s Book* (London: Wolfe Publishing Ltd., 1967), pp.17, 23.

75. The Editors of Slimmer Magazine, *Let’s Start to Slim* (London: Ward Lock Ltd., 1977), p.10.

76. See also Marguerite Patten, *Marguerite Patten’s Slimmers Diary* (London, Collins, 1976 and subsequent editions, 1978, 1979), which uniformly assumed that the reader will be self-weighing.

77. Compare “Miscellany,” *Marguerite Patten’s Manchester Guardian*, September 29, 1948, mocking the idea that “the ideal Englishman is the rationed citizen of the (new) hungry forties,” with David Langdon, “Phew! You are an Obese Sixteen Stone Ten and a National Disgrace,” *Sunday Mirror*, April 6, 1969, British Cartoon Archive, University of Kent (BCA) <https://archive.cartoons.ac.uk/Record.aspx?src=CalmView.Catalog&id=LA0007B&pos=1> (February 24, 2018).

78. See Jane Hand, “‘Tucking in Your Tummy Isn’t the Answer!’: Visualising Obesity as a Public Health Concern in 1970s and 1980s Britain,” in Mark Jackson and Martin Moore (eds.), *Balancing the Self: Medicine, Politics and the Regulation of Health in the Twentieth Century* (Manchester: Manchester University Press, 2020).

79. Mass Observation Ltd., *Background Study on Slimming* (London: Mass-Observation Limited, 1968), pp.9, 6.

80. Ibid., p.11.

81. Hand, “Marketing Health Education” (note 60).

82. Mass Observation Ltd., *Study on Slimming*, p.13 (note 79).

83. J. C. McKenzie, “Profile on Slimmers,” *Commentary: The Journal of the Market Research Society* 9(2) (1967): 77–83, quoted in Mass Observation Ltd., “Study on Slimming,” p.27 (note 79).

84. Visual examples include Ronald Giles, “I’d like to get within catapult range of that school medical officer who says there are too many Billy Bunters these days.” *Daily Express*, August 25, 1959; Stanley Franklin, “This is Clare before we put her on a diet,” *Daily Mirror*, May 1964; Ronald Giles, “‘Yaroo! Back come all the Fanny Hills and Lady Chatterleys – out go all the Billy Bunters,’” *Sunday Express*, February 15, 1970; all BCA <www.cartoons.ac.uk/> (February 24, 2018).

85. Instead, as Berridge and others have demonstrated, alcohol and cigarettes became the first targets of the newly mediatized health promotions lobby. Fatty and sugary foods only began to attract similar (but far less interventionist) attention in the mid 1970s.

86. Hand, “Tucking in Your Tummy” (note 78).

87. E.g. Charlotte Biltekoff, Jessica Mudry, Aya H. Kimura, Hannah Landecker, and Julie Guthman, “Interrogating Moral and Quantification Discourse in Nutritional Knowledge,” *Gastronimica* 14(3) (2014): 17–26, 24; Marcia Meldrum, “‘Simple Methods’ and ‘Determined Contraceptors’: The Statistical Evaluation of Fertility Control, 1957-1968,” *Bulletin of the History of Medicine* 70(2): (1996): 266–95; Rebecca J. Williams, “Revisiting the Khanna Study: Population and Development in India, 1953-60,” unpublished PhD dissertation, University of Warwick, 2014.

88. See TNA INF13/194 “Seven Rules of Health,” <www.nationalarchives.gov.uk/pathways/citizenship/brave_new_world/docs/health_poster.htm> (February 24, 2018) and TNA BN10/32 “The Right Foods to Eat,” a poster of a similar vintage, which deployed pictures of food staples to illustrate “three rules … to keep you in good health.”

89. Hand, “Tucking in your Tummy,” pp.1–2 (note 78); Bufton and Berridge, “Post-War Nutrition Science,” pp.216–17 (note 45).

90. On Quetelet, Garabed Eknoyan, “Adolphe Quetelet (1796-1874) – the Average Man and Indices of Obesity,” *Nephrology Dialysis Transplantation* 23(1) (2008): 47–51; Gustav Jahoda, “Quetelet and the Emergence of the Behavioral Sciences,” *SpringerPlus* 4(1) (2015): 473; for a wider history, Theodor Porter, *The Rise of Statistical Thinking, 1820–1900* (Princeton: Princeton University Press, 1986).

91. Isabel Fletcher, “Defining an Epidemic: The Body Mass Index in British and American Obesity Research 1960-2000,” *Sociology of Health and Illness* 36(3) (2014): 338–53, fn. 23. On flaws in BMI, Emily Yates-Doerr, “The Mismeasure of Obesity,” in Megan B. McCullough and Jessica A. Hardin (eds.), *Reconstructing Obesity: The Meaning of Measures and the Measure of Meanings* (New York: Berghahn Books, 2013), pp.49–72; Anne E. Becker, “Resocializing Body Weight Obesity and Health Agency,” in Megan B. McCullough and Jessica A. Hardin (eds.), *Reconstructing Obesity: The Meaning of Measures and the Measure of Meanings* (New York: Berghahn Books, 2013), pp.27–48, 29.

92. Fletcher, “Defining an Epidemic,” 343–44 (note 91); Yates-Doerr, “Mismeasure of Obesity,” pp.52–4 (note 91).

93. Yates-Doerr, “Mismeasure of Obesity,” pp.53–4 (note 91).

94. Andrew Veitch, “Two Nation Divide in Health as Well as Wealth,” *The Guardian*, April 27, 1987.

95. Keith Thomas, “Letter to the Editor: Slim Pickings,” *The Guardian*, March 1, 1976.

96. For a near-contemporary actor’s account, Philip James, “Cantor Lectures. The Implications of a Change of Diet. 1. Dietary Reform: An Individual or National Response,” *RSA Journal* 136(5382) (1988): 373–87.

97. Sylvia J. Darke, “Monitoring the Nutritional Status of the UK Population,” *Proceedings of the Nutrition Society* 36 (1977): 235–40, 240.

98. Boyd Swinburn, “Why Are Governments Abdicating from Dealing with the Obesity Crisis?” in David Haslam, Arya Sharma, and Carel le Roux (eds.), *Controversies in Obesity* (London: Springer, 2014), pp.23–29.

99. Ian Mather, “Counting the Losses”, *Daily Mail*, March 12, 1976.

100. “Slim Line Tonic for the NHS,” *The Guardian*, September 1, 1976.

101. Darke, “Monitoring the Nutritional Status,” 240 (note 97).

102. Rosemary Collins, “How Many Greasy Chips Constitute a Health Hazard?” *The Guardian*, April 23, 1983. On the role of the food industry in health education, see Hand, “Marketing Health Education” (note 60). For a counter-narrative of U.S. guidelines, see Robert Kuczmarski and Katherine Flegal, “Criteria for Definition of Overweight in Transition: Background and Recommendations for the United States,” *American Journal of Clinical Nutrition* 72 (2000): 1074–81 for the slow transition to BMI by Federal agencies, which featured in official health guidance *for the public* for the first time in 1995.

103. Richard Adler, “How Heart Disease Can Be Beaten,” *Financial Times*, June 22, 1985.

104. The UK would not be alone in drawing such connections between population weights and national identity and status. See Annemarie Jutel, “Does Size Really Matter? Weight and Values in Public Health,” *Perspectives in Biology and Medicine* 44(2): (2001): 283–96.

105. Alan Pike, “The Challenge of Changing the Habits of Millions,” *Financial Times*, January 15, 1988.

106. E.g. Richard Woodman, “Special Report: Heart Stopping,” *Daily Mail*, July 5, 1994, where readers were gloomily told, “Almost all nations are faring better than the UK …We are a nation at risk.”

107. “Fat: A Matter of Judgement,” *The Times*, September 4, 2003. See also Annabel Ferriman, “Health: Mirror, Mirror…,” *The Guardian*, April 21, 1998.

108. N. J. C. Grant and M. E. Ruttley, “Letters: GPs’ New Contract,” *The Times*, October 24, 1989. On the GP contract and changes to preventive care, see Simon J. Williams, Michael Calnan, Sarah L. Cant, and Joanne Coyle, “All Change in the NHS? Implications of the NHS Reforms for Primary Care Prevention,” *Sociology of Health and Illness* 15(1) (1993): 43–67, 45.

109. Williams et al., “All Change?” p.45 (note 108).

110. I searched the digital archives of these three newspapers via a combination of the Proquest advanced search tool and their own proprietary tools (where available), using the term “Body mass index,” hand-weeding for duplicates and irrelevant or non-sense results.

111. E.g. “We Changed Our Lives: So Can You,” *Daily Mail*, January 6, 1997; Jenny Hope, “One Briton in Two is Warned over Weight,” *Daily Mail*, May 11, 1998.

112. “Testing Time,” *The Guardian*, May 11, 1999.

113. Michael Hann, “Jonah Lomu is Fat,” *The Guardian*, September 17, 2002.

114. Chris Mihill, “Obesity Costs Early Death and 200m,” *The Guardian*, July 18, 1994; “£200m Bill for the Fat of the Land,” *Daily Mail*, July 18, 1994.

115. Marie Woolf, “Overweight Patients May Have to Diet to See Doctor,” *The Independent*, June 3, 2003.

116. Jenny Hope, “One Briton in Two is Warned over Weight,” *Daily Mail*, May 11, 1998; Beezy Marsh, “Heavy Cost of Obesity,” *Daily Mail*, November 1, 1999.

117. J. Monaham, “Why Being Obese is Bad for the Country,” *The Guardian*, February 13, 2001.

118. E.g. Lorna Duckworth, “Growing Problem of Obesity Costs £2.5bn a Year,” *The Independent*, February 15, 2001.

119. E.g. Sarah Boseley, “Food Industry Blamed for Surge in Obesity,” *The Guardian*, September 13, 2002; Duckworth, “Growing Problem of Obesity” (note 118) (which includes dramatic comparative charts).

120. Jenny Hope, “The Bottom Line is, Healthy Ladies are Pear-shaped,” *Daily Mail*, December 7, 1999.

121. Robert Beaglehole, quoted in Sarah Boseley, “Sick to Our Stomachs’, *The Guardian*, September 25, 2004.

122. Department of Health, *Choosing Health? A Consultation on Action to Improve People’s Health* HM Government, March 3, 2004, p.20 <https://webarchive.nationalarchives.gov.uk/20050404201427/http://www.dh.gov.uk/Consultations/ResponsesToConsultations/ResponsesToConsultationsDocumentSummary/fs/en?CONTENT_ID=4106017&chk=utuMSG> (March 29, 2018). The survey received 2,230 submissions, substantially more than two previous consultations on “Choosing Activity” and “Choosing a Better Diet,” which elicited 283 and 218 submissions respectively (see Department of Health, *Choosing Health: Making Health Choices Easier* HM Government, November 12, 2004, p.184, <https://webarchive.nationalarchives.gov.uk/20041206180054/http://www.dh.gov.uk/PublicationsAndStatistics/Publications/PublicationsPolicyAndGuidance/PublicationsPolicyAndGuidanceArticle/fs/en?CONTENT_ID=4094550&chk=aN5Cor> (March 29, 2018).

123. Tony Blair, “Foreword,” *Choosing Health?* p.3 (note 122).

124. John Reid, “Foreword,” *Choosing Health?* p.6 (note 122).

125. Julie Cream, David Maguire, and Ruth Robertson, “How Have Public Attitudes to the NHS Changed in the Past Three Decades,” King’s Fund, February 1, 2018, <www.kingsfund.org.uk/publications/how-have-public-attitudes-to-nhs-changed> (March 29, 2018).

126. “How to Beat Fat,” *The Times*, November 25, 2004; and for comparison, James Le Fanu, “Quack Medicine by Nanny Bottomley,” *The Times*, November 16, 1993.

